# Molecular epidemiology, antimicrobial resistance, virulence profiling, and pathogenicity of bovine Pasteurella multocida isolates from Hebei Province, China: Dominance of the A:L3-ST79 clonal lineage

**DOI:** 10.14202/vetworld.2026.3915-3931

**Published:** 2026-09-07

**Authors:** Yuntai Li, Jianjian Hu, Jialei Pei, Xuqiang Zhang, Li Chen, Zhiqiang Zhang, Yonghui Li, Xiaochang Pang, Ziteng Lu, Qiumei Shi, Tonglei Wu

**Affiliations:** 1 Hebei Provincial Key Laboratory of Preventive Veterinary Medicine, Hebei Normal University of Science and Technology, Qinhuangdao 066004, PR China.; 2 Shijiazhuang Animal Disease Prevention and Control Center, Shijiazhuang 050000, PR China.; 3 The Second Hospital of Qinhuangdao, Qinhuangdao 066600, PR China.

**Keywords:** antimicrobial resistance, bovine pasteurellosis, capsular typing, molecular epidemiology, multilocus sequence typing, Pasteurella multocida, pathogenicity, virulence genes

## Abstract

**Background and Aim:**

*Pasteurella multocida* is an important bacterial pathogen associated with bovine respiratory disease and septicemia, causing substantial economic losses to the cattle industry worldwide. Increasing antimicrobial resistance has complicated disease management, yet comprehensive information on the molecular epidemiology, virulence characteristics, antimicrobial resistance, and pathogenicity of bovine *P. multocida* in Hebei Province, China, remains limited. This study aimed to characterize the circulating molecular lineages, virulence profiles, antimicrobial resistance patterns, and pathogenicity of bovine *P. multocida* isolates to support evidence-based surveillance, disease control, and rational antimicrobial use.

**Materials and Methods:**

Between 2023 and 2025, 162 clinical samples were collected from diseased cattle in seven prefecture-level cities of Hebei Province. *P. multocida* isolates were identified using conventional bacteriology and *kmt* gene polymerase chain reaction (PCR). Capsular and lipopolysaccharide (LPS) typing, RIRDC multilocus sequence typing, PCR detection of 15 virulence genes and 12 antimicrobial resistance genes, antimicrobial susceptibility testing against 14 antimicrobial agents using the Kirby–Bauer disk diffusion method, and pathogenicity evaluation in Kunming mice by determining the median lethal dose were performed.

**Results:**

Sixty-four *P. multocida* isolates were recovered, yielding an overall isolation rate of 39.5%. Capsular type A (95.3%) and LPS genotype L3 (87.5%) predominated, with the A:L3 genotype accounting for 84.4% of all isolates. Sequence type ST79 represented 79.7% of all isolates and 94.4% of A:L3 isolates, demonstrating the dominance of the A:L3-ST79 clonal lineage. Twelve virulence-associated genes, including *pfhA, tbpA, pmHAS, tadD,* *ompA*, *oma87*, *fimA*, *nanH*, *exbB*, *exbD*, *tonB*, and *hgbA*, were detected in 96.9%–100% of isolates, whereas *toxA* was absent. The macrolide resistance gene *mphE* (92.2%) and sulfonamide resistance gene *sul2* (87.5%) were the predominant resistance determinants. Multidrug resistance was identified in 95.3% of isolates, although high susceptibility was retained to doxycycline, enrofloxacin, ciprofloxacin, ceftiofur, and florfenicol. Representative A:L3-ST79 isolates exhibited high pathogenicity in mice, with median lethal dose values ranging from 8.06 × 10¹ to 6.44 × 10³ colony-forming units.

**Conclusion:**

Bovine *P. multocida* isolates circulating in Hebei Province are dominated by a highly virulent and multidrug-resistant A:L3-ST79 clonal lineage with distinct molecular epidemiological characteristics. These findings provide a comprehensive baseline for molecular surveillance, vaccine development, antimicrobial stewardship, and targeted prevention and control strategies against bovine pasteurellosis in northern China.

## INTRODUCTION

*Pasteurella multocida* is a Gram-negative coccobacillus with bipolar staining characteristics [1]. It widely colonizes the upper respiratory mucosa of various wild animals, livestock, and poultry and is an important opportunistic zoonotic pathogen [2, 3]. Under stress conditions such as transportation, abrupt climate changes, and high stocking density, *P. multocida* can breach the host mucosal barrier and cause local or systemic infections, clinically manifesting as hemorrhagic septicemia, pneumonia, atrophic rhinitis, fowl cholera, and other diseases, resulting in substantial economic losses to the global livestock industry [4, 5].

Bovine pasteurellosis, also known as bovine hemorrhagic septicemia, is an acute or chronic infectious disease caused by *P. multocida*. Acute cases are typically characterized by high fever, mucosal hemorrhage, dyspnea, and high mortality [6]. In recent years, the rapid scale-up and intensification of cattle farming in China have increased stocking density, and bovine respiratory disease and septicemia caused by *P. multocida* have become important constraints on healthy cattle production [7].

According to capsular polysaccharide composition, *P. multocida* can be divided into five capsular serogroups: A, B, D, E, and F [8]. Based on differences in the lipopolysaccharide (LPS) biosynthesis gene cluster, it can also be classified into eight LPS genotypes, L1-L8 [9]. Among these, capsular type A strains are closely associated with respiratory infections in cattle, pigs, and poultry [7, 10, 11]. Previous studies have shown that capsular type A is the predominant serotype among *P. multocida* isolates associated with bovine respiratory disease, with A:L3 being a major circulating type [11, 12].

Molecular epidemiology is essential for elucidating pathogen transmission routes, population structure, and evolutionary characteristics [13]. Multilocus sequence typing (MLST), based on sequence variation in housekeeping genes, enables accurate tracing of clonal lineages and transmission patterns [14]. Capsular typing and LPS typing further clarify serological features and provide a basis for epidemiological surveillance [9, 15]. The pathogenic mechanism of *P. multocida* is complex and depends on the coordinated action of multiple virulence factors, including capsular polysaccharide, outer membrane proteins (OmpA and Oma87), fimbriae (FimA), iron acquisition systems (ExbB, ExbD, and TonB), neuraminidases (NanB and NanH), and dermonecrotic toxin (ToxA) [2, 16]. Strains from different serotypes and geographic origins may show substantial differences in virulence gene carriage, which directly affects pathogenicity and host adaptation [17].

Antimicrobial agents remain the primary approach to the clinical treatment of bovine pasteurellosis. However, with the long-term and widespread use of antibiotics in animal production, antimicrobial resistance in *P. multocida* has become increasingly severe [18]. Resistance to ampicillin, tetracyclines, macrolides, and sulfonamides has been reported, and some strains display multidrug-resistant or even extensively drug-resistant phenotypes [19]. The underlying mechanisms mainly include beta-lactamase production, overexpression of efflux pumps, target-site mutations, and horizontal transfer of resistance genes [18]. In cattle production in China, tetracyclines, macrolides, and sulfonamides are frequently and widely used, further promoting the dissemination of related resistance genes in *P. multocida* and posing a serious challenge to effective clinical treatment [20]. Hebei Province is an important dairy and beef cattle production region in northern China, with a large breeding scale and broad geographic distribution across diverse ecological conditions and farming systems [21]. However, systematic studies on bovine *P. multocida* in Hebei Province remain limited, particularly those integrating circulating serotypes, predominant clonal lineages, virulence gene profiles, resistance phenotypes and genotypes, and pathogenicity.

Although *P. multocida* has been studied in various regions of China and globally, systematic data integrating capsular/LPS typing, RIRDC MLST, comprehensive virulence gene profiling, phenotypic and genotypic antimicrobial resistance analysis, and *in vivo* pathogenicity assessment of bovine isolates from Hebei Province—a major cattle-producing region in northern China, are lacking. Previous studies have often focused on single aspects (e.g., serotyping or resistance only) and rarely examined the dominant clonal lineages in relation to virulence, resistance, and pathogenicity under local farming conditions. There is also limited information on LPS genotype-associated differences in virulence gene carriage and the persistence of specific resistance genes linked to historical antimicrobial use patterns in this region.

This study aimed to (1) determine the capsular and LPS genotypes and RIRDC MLST profiles of bovine *P. multocida* isolates from Hebei Province, (2) characterize the distribution of key virulence genes and antimicrobial resistance genes, (3) evaluate phenotypic antimicrobial susceptibility patterns, and (4) assess the pathogenicity of the predominant clonal lineage using a mouse model, thereby providing a scientific basis for molecular surveillance, targeted prevention and control, vaccine development, and rational antimicrobial therapy for bovine pasteurellosis in northern China.

## MATERIALS AND METHODS

### Ethical approval

The animal experiments were reviewed and approved by the Institutional Animal Care and Use Committee (IACUC) of Hebei Normal University of Science and Technology, Qinhuangdao, China, and were conducted in accordance with the Regulations for the Administration of Laboratory Animals issued by the Hebei Provincial Department of Science and Technology (HPDST 2020-17) and internationally accepted guidelines for the care and use of laboratory animals. All procedures involving Kunming mice (SPF (Beijing) Biotechnology Co., Ltd., Beijing, China) were performed under specific-pathogen-free conditions by trained personnel. Animals were monitored throughout the study, and humane endpoints were applied to minimize pain and distress. Mice exhibiting severe clinical signs, including marked respiratory distress, inability to access food or water, or prostration, were humanely euthanized under isoflurane anesthesia followed by cervical dislocation.

Clinical samples from diseased cattle were collected as part of routine veterinary diagnostic investigations with the permission of farm owners and did not involve additional invasive procedures beyond standard veterinary practice. Sample collection was performed by licensed veterinarians in accordance with institutional animal welfare and biosafety requirements.

### Study period and location

From March 2023 to April 2025, clinical samples were collected from diseased cattle in seven prefecture-level cities of Hebei Province (Qinhuangdao, Chengde, Tangshan, Shijiazhuang, Hengshui, Xingtai, and Handan).

### Study design

This cross-sectional study involved isolation, molecular characterization, antimicrobial susceptibility testing, and *in vivo* pathogenicity assessment of *P. multocida* isolates.

### Sample sources and bacterial culture conditions

A total of 162 clinical samples were collected from diseased cattle in seven prefecture-level cities in Hebei Province, including Qinhuangdao, Chengde, Tangshan, Shijiazhuang, Hengshui, Xingtai, and Handan. The samples comprised nasal swabs from cattle with apparent respiratory symptoms and lung tissues from cattle that died of pneumonia or septicemia. All samples were transported under refrigeration and subjected to bacterial isolation immediately upon arrival. During isolation and purification, bacterial isolates were cultured on sheep blood agar plates (Wenzhou Kangtai Biotechnology Co., Ltd., Wenzhou, China). Purified *P. multocida* isolates were cultured on Brain Heart Infusion (BHI) agar or in BHI (Qingdao Hope Biotechnology Co., Ltd., Qingdao, China) broth at 37 °C, and the cultures were preserved in 25% (v/v) glycerol at −80 °C for long-term storage.

### Isolation and identification of *P. multocida*

Clinical samples from dairy and beef cattle were streaked onto blood agar plates for bacterial isolation. Dominant grayish-white, drop-like colonies were selected and purified. Purified isolates were subjected to Gram staining (Beijing Boao Kuoda Biotechnology Co., Ltd., Beijing, China) and identified by polymerase chain reaction (PCR) amplification of the *kmt* gene, following methods described by Townsend *et al*. [22]. Primer (Sangon Biotech Co., Ltd., Beijing, China) sequences are listed in Table 1. Bacterial genomic DNA was prepared by boiling. Briefly, 1 mL of logarithmic-phase *P. multocida* culture was centrifuged at 12,000 r/min for 3 min, and the supernatant was discarded. The pellet was resuspended in an equal volume of sterile saline, heated in a dry bath at 100 °C for 10 min, and centrifuged again; the supernatant was used as the DNA template. The PCR reaction mixture (20 μL) contained 10 μL of 2× Taq PCR MasterMix (CW Biotech Co., Ltd., Taizhou, China), 1 μL each of forward and reverse primers for the *kmt* gene, 2 μL of DNA template, and 6 μL of double-distilled water (ddH₂O), with a negative control included. The PCR amplification program was as follows: initial denaturation at 94 °C for 5 min; 30 cycles of denaturation at 94 °C for 30 s, annealing at 30 s (annealing temperatures are listed in Table 1), and extension at 72 °C for 1 min; and a final extension at 72 °C for 10 min. PCR products were detected by electrophoresis on 1.5% agarose gels. The isolates were identified as *P. multocida* through a combination of microbiological morphology and molecular biology methods.

### Identification of capsular serotypes and LPS genotypes

Primers for capsular and LPS typing of *P. multocida* were synthesized according to published methods. Capsular multiplex PCR [15] and LPS multiplex PCR [9] were used to determine capsular types and LPS genotypes, respectively. Primer (Sangon Biotech Co., Ltd.) sequences are listed in Table 1. The capsular multiplex PCR reaction was carried out in a 20 μL mixture containing 10 μL of 2× Taq PCR MasterMix (CW Biotech Co., Ltd.), 0.4 μL each of five primer pairs (forward and reverse), 2 μL of DNA template, and 4 μL of ddH₂O. The LPS multiplex PCR reaction was carried out in a 25 μL mixture containing 10 μL of 2× Taq PCR MasterMix, 0.5 μL each of eight primer pairs (forward and reverse), 2 μL of DNA template, and 5 μL of ddH₂O. The PCR amplification program was identical to that described above, with annealing temperatures listed in Table 1. A negative control was included in each assay. Ambiguous results were confirmed by single-pair PCR.

**Table 1:** Primers used for Pasteurella multocida identification, capsular typing, lipopolysaccharide typing, and RIRDC multilocus sequence typing.

**Primer name**	**Primer sequence (5′–3′)**	**Annealing temperature (°C)**	**Amplicon size (bp)**
*kmt*	F: ATCCGCTATTTACCCAGTGG R: GCTGTAAACGAACTCGCCAC	55	460
Capsular serotype A (type A)	F: TGCCAAAATCGCAGTCAG R: TTGCCATCATTGTCAGTG	53	1044
Type B	F: CATTTATCCAAGCTCCACC R: GCCCGAGAGTTTCAATCC	53	760
Type D	F: TTACAAAAGAAAGACTAGGAGCCC R: CATCTACCCACTCAACCATATCAG	53	657
Type E	F: TCCGCAGAAAATTATTGACTC R: GCTTGCTGCTTGATTTTGTC	53	511
Type F	F: AATCGGAGAACGCAGAAATCAG R: TTCCGCCGTCAATTACTCTG	53	851
L1	F: ACATTCCAGATAATACACCCG R: ATTGGAGCACCTAGTAACCC	53	1307
L2	F: CTTAAAGTAACACTCGCTATTGC R: TTTGATTTCCCTTGGGATAGC	53	810
L3	F: TGCAGGCGAGAGTTGATAAACCATC R: CAAAGATTGGTTCCAAATCTGAATGGA	53	474
L4	F: TTTCCATAGATTAGCAATGCCG R: CTTTATTTGGTCTTTATATATACC	53	550
L5	F: AGATTGCATGGCAGAAATGGC R: CAATCCTCGTAAGACCCCC	53	1175
L6	F: TCTTTATAATTATACTCTCCCAAGG R: AATGAAGGTTTAAAAGAGATAGCTGGAG	53	668
L7	F: CCTATATTTATATCTCCTCCCC R: CTAATATATAAACCATCCAACGC	53	931
L8	F: GAGAGTTACAAAAATGATCGGC R: TCCTGGTTCATATATAGGTAGG	53	255
*adk*	F: TTTTTCGTCCCGTCTAAGC R: GGGGAAAGGGACACAAGC	54	570
*est*	F: TCTGGCAAAAGATGTTGTCG R: CCAAATTCTTGGTTGGTTGG	54	641
*pmi*	F: TGCCTTGAGACAGGGTAAGC R: GCCTTAACAAGTCCCATT CG	54	739
*zwf*	F: AATCGGTCGTTTGACTGAGC R: TGCTTCACCTTCAACTGTGC	54	808
*mdh*	F: ATTTCGGGATCAGGGTTAGC R: GGAAAACCGGTAATGGAAGG	54	620
*gdh*	F: ATCGACTTCTTCCGCAGACC R: GCGGGTGATATTGGTGTAGG	54	702
*pgi*	F: ACCACGCTATTTTTGGTTG R: ATGGCACAACCTCTTTCACC	54	784

### RIRDC MLST typing

According to the sequences provided in the PubMLST database, seven housekeeping genes (*adk*, *est*, *pmi*, *zwf*, *mdh*, *gdh*, and *pgi*) used in the RIRDC MLST scheme for *P. multocida* were selected. Primer (Sangon Biotech Co., Ltd.) sequences are shown in Table 1. Each housekeeping gene was amplified by PCR [23]. The PCR reaction mixture (50 μL) contained 25 μL of 2× Taq PCR MasterMix (CW Biotech Co., Ltd.), 1 μL each of forward and reverse primers for each individual housekeeping gene, 2 μL of DNA template, and 21 μL of ddH₂O, with a negative control included. The PCR amplification program was identical to that described above, with annealing temperatures listed in Table 1. PCR products with clear specific bands were sent to Sangon Biotech (Shanghai) Co., Ltd. for bidirectional sequencing and assembly. The assembled sequences of each housekeeping gene were submitted to the *P. multocida* RIRDC MLST database for comparison to obtain the corresponding allele numbers, and the RIRDC MLST type of each isolate was subsequently determined based on the combination of the seven allele numbers.

### Detection of virulence genes

Based on published primer sequences, 15 virulence genes in nine categories of *P. multocida* [17, 24] were detected, including outer membrane protein genes (*ompA* and *oma87*), fimbrial gene (*fimA*), filamentous hemagglutinin gene (*pfhA*), neuraminidase genes (*nanB* and *nanH*), iron acquisition-related genes (*exbB*, *exbD*, *tonB*, and *tbpA*), hemoglobin-binding protein genes (*hgbA* and *hgbB*), hyaluronidase gene (*PmHAS*), dermonecrotic toxin gene (*toxA*), and adhesin gene (*tadD*). Primer (Sangon Biotech Co., Ltd.) sequences are listed in Table 2. The PCR reaction mixture (20 μL) contained 10 μL of 2× Taq PCR MasterMix (CW Biotech Co., Ltd.), 1 μL each of forward and reverse primers, 2 μL of DNA template, and 6 μL of ddH₂O, with a negative control included. The PCR amplification program was identical to that described above, with annealing temperatures listed in Table 2.

**Table 2:** Primers used for detection of Pasteurella multocida virulence genes.

**Primer name**	**Primer sequence (5′–3′)**	**Annealing temperature (°C)**	**Amplicon size (bp)**
*ompA*	F: TTCCGCCGTCAATTACTCTG R: CATAAACAGATTGACCGAAAACG	53	201
*oma87*	F: GGCAGCGAGCAACAGATAACG R: TGTTGTCAAATGTCGGGTGA	55	838
*fimA*	F: CCATCGGATCTAAACGACCTA R: AGTATTAGTTCCTGCGGGTG	55	866
*pfhA*	F: TTCAGAGGGATCAATCTTCG R: AACTCCAGTTGTTTTGTCG	53	286
*nanB*	F: CATTGCACCTCAACACCTCT R: GGACACTGATTGCCCTGAA	53	555
*nanH*	F: GTGGGAAACGGGGAATTGTGA R: GTGGGAAACGGGGAATTGTGA	55	287
*exbB*	F: TTGGCTTGTGATTGAACAGC R: TGCAGGAATGGCGACTAAA	55	283
*exbD*	F: CGTTCTGATTACAGCCTCTT R: AACGAAATCTTGGAAACTGG	53	247
*tonB*	F: CGACGGTGAAACCTGGAGCCA R: CCGAGCGATAAAGCATTGACT	55	261
*tbpA*	F: TGGTTGGAAACGGTAAAGC R: TAACGGTGTACGGAAA AAGCC	53	728
*hgbA*	F: TGGCGGATAGTCATCATCAAG R: CCAAAGAAACCACTACACCCA	53	419
*hgbB*	F: ACCGCGTTGGAAATTATGATTG R: CATTGAGTACGGCTTGACAT	53	788
*PmHAS*	F: TCAATGTTTTGCGATAGTCCGTTAG R: TGGCG AATGATCGGTGATAGA	55	430
*toxA*	F: CTTAGATGAGCGACAAAGG R: GAATGCCACACCTCTATAG	50	864
*tadD*	F: TCTACCCATTCTCAGCAAGGC R: ATCATTTCGGGCATTCACC	55	416

### Detection of antimicrobial resistance genes

Based on published primer sequences, 12 resistance genes in eight antimicrobial classes [25, 26] were detected, including resistance genes of aminoglycosides (*strA* and *strB*), resistance genes of macrolides (*ermA* and *mphE*), resistance genes of tetracyclines (*tetA*), resistance genes of quinolones (*qnrA* and *qnrB*), beta-lactams (*blaTEM* and *blaOXA*), resistance genes of lincosamides (*lnuA*), resistance genes of phenicols (*floR*), and resistance genes of sulfonamides (*sul2*). The PCR reaction mixture and amplification program were identical to those used for virulence gene detection. Primer (Sangon Biotech Co., Ltd.) sequences and annealing temperatures are listed in Table 3.

**Table 3:** Primers used for detection of Pasteurella multocida antimicrobial resistance genes.

**Primer name**	**Primer sequence (5′–3′)**	**Annealing temperature (°C)**	**Amplicon size (bp)**
*strA*	F: AAACGAGGCTGGAAAAGG R: ATCAACTGGCAGGAGGAA	53	681
*strB*	F: GTTGCTCCTCTTCTCCATC R: CACCTTTTCCAGCCTCGT	53	722
*ermA*	F: GTTCAAGAACAATCAATACAGAG R: GGATCAGGAAAAGGACATTTTTAC	50	421
*mphE*	F: ACAAAGATAGCCCGAAATAC R: TGACCAATCAATAACGCC	53	314
*tetA*	F: GTGAAACCCAACATACCCC R: GAAGGCAAGCAGGATGTAG	53	888
*qnrA*	F: CAAGAGGATTTCTCACGCCAG R: AATCCGGCAGCACTATTACTCC	55	628
*qnrB*	F: AGCGGCACTGAATTTATCGG R: CGCAATGTGTGAAGTTTGCT	55	418
*blaTEM*	F: GAGTATTC AACATTTTCGT R: ACCAATGCTTAATCAGTGA	46	857
*blaOXA*	F: GCAGCGCCAGTGCATCAAC R: CCGCATCAAATGCCATAAGTG	55	198
*lnuA*	F: GCATGTTATTGATTTTAAATT R: GCTTAGGAGGGATAAAATGAA	53	500
*floR*	F: ATTTTATCTCTCCCTGTCGTTCC R: TCCCGCGAACAATGCTGAGACTAT	53	982
*sul2*	F: CCGGTCTCTCGCTCTCGAACAAGTTA R: CTCGGTGTGTGTGCGGGATGGAAGAGT	55	509

### Antimicrobial susceptibility testing

The susceptibility of all isolates to 14 antimicrobial agents (Hangzhou Microbiology Reagent Co., Ltd., China) from eight classes was determined by the Kirby-Bauer disk diffusion method in accordance with the Clinical and Laboratory Standards Institute (CLSI) guidelines and the Chinese health industry standard WS/T 639-2018. *Escherichia coli ATCC 25922* was used as the quality control strain. After incubation at 37 °C for 18 h, the results were interpreted in accordance with CLSI and WS/T 639-2018 criteria. Isolates resistant to three or more antimicrobial classes were defined as multidrug-resistant isolates.

### Animal pathogenicity assay

Three representative A:L3-ST79 isolates from different geographic regions were selected to determine the median lethal dose (*LD*₅₀) in Kunming mice (SPF (Beijing) Biotechnology Co., Ltd.) and to evaluate pathogenicity. The isolates were inoculated into BHI (Qingdao Hope Biotechnology Co., Ltd.) medium and cultured at 37 °C with shaking at 180 r/min for 8 h, followed by viable colony counting. The bacterial suspensions were washed twice with sterile saline and diluted to five concentration gradients ranging from 1 × 10⁶ to 1 × 10² colony-forming units (CFU)/mL. A total of 80 six-week-old female Kunming mice were randomly divided into 16 groups, including one control group and 15 experimental groups (five groups each for isolates QA-1, TA-1, and SA-1). Each mouse in the challenge groups received an intraperitoneal injection of 0.2 mL of bacterial suspension, whereas the control group received an equal volume of sterile saline. Mice were observed continuously for 7 days. Individuals exhibiting severe respiratory distress, inability to access food or water, or prostration were euthanized humanely by cervical dislocation under isoflurane anesthesia, and the time of death was recorded. The *LD*₅₀ values were calculated using the Karber method.

### Statistical analysis

Statistical analyses were performed using GraphPad Prism 11.0.0. Fisher's exact test in 2×2 contingency tables was used to analyze the association between A:L3 and ST79, as well as differences in gene carriage and antimicrobial susceptibility among isolates. A *p*-value of < 0.05 was considered statistically significant. All percentages presented in this study were rounded to one decimal place.

## RESULTS

### Isolation and identification of *P. multocida*

A total of 64 purified bacterial isolates were obtained from 162 clinical samples collected from diseased cattle in seven cities of Hebei Province after isolation on sheep blood agar plates. The isolates formed typical grayish-white, moist, smooth, translucent, drop-like colonies without hemolysis on blood agar plates (Figure 1A). Gram staining showed Gram-negative coccobacilli with clear bipolar staining (Figure 1B). All isolates yielded a specific PCR band of approximately 460 bp for the *kmt* gene, consistent with the expected fragment size (Figure 1C). Based on colony morphology, staining characteristics, and molecular identification, all 64 isolates were confirmed as *P. multocida*.

**Figure 1:**
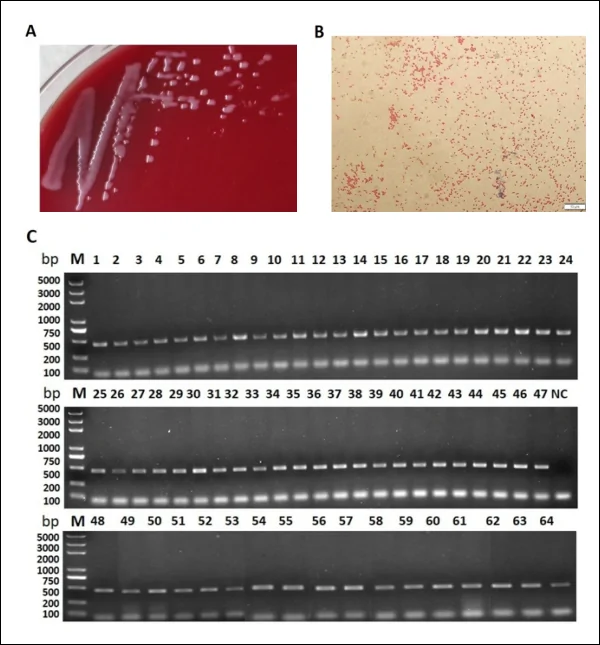
Isolation and identification of *Pasteurella multocida*. (A) Colony morphology; (B) Gram staining at 1000× magnification; (C) Polymerase chain reaction identification of the isolates; lanes 1–64, isolates; NC = Negative control.

### Geographic distribution of isolates

A total of 162 clinical samples were collected from diseased cattle, yielding 64 *P. multocida* isolates, for an overall isolation rate of 39.5% (Table 4). Samples were collected from seven prefecture-level cities in Hebei Province: Qinhuangdao, Chengde, Tangshan, Shijiazhuang, Hengshui, Xingtai, and Handan. Among them, Qinhuangdao contributed 59 samples, from which 27 isolates were obtained, yielding an isolation rate of 45.8% and accounting for 42.2% of all isolates, making it the major source region (Figure 2). Nine isolates were obtained from Shijiazhuang (42.9%), five from Handan (41.7%), three from Hengshui (37.5%), nine from Xingtai (36.0%), seven from Tangshan (30.4%), and four from Chengde (28.6%). The isolation rates ranged from 28.6% to 45.8% across regions, indicating that *P. multocida* was distributed in both northern and southern Hebei Province.

**Table 4:** Sample sources and Pasteurella multocida isolation rates.

**Region**	**No. of samples**	**No. of ** ***P. *** ***multocida*** ** isolates**	**Isolation rate (%)**
Qinhuangdao	59	27	45.8
Chengde	14	4	28.6
Tangshan	23	7	30.4
Shijiazhuang	21	9	42.9
Hengshui	8	3	37.5
Xingtai	25	9	36.0
Handan	12	5	41.7
Total	162	64	39.5

The isolation rate (%) was calculated as (number of isolates/number of samples) × 100% and rounded to one decimal place.

### Capsular serotyping and LPS genotyping

Capsular typing of the 64 *P. multocida* isolates showed that 61 isolates (95.3%) belonged to capsular type A, which was the overwhelmingly predominant serotype (Table 5, Figure 3). Types B, D, and F were each represented by one isolate (1.6% each), whereas type E was not detected. LPS genotyping showed that L3 was the predominant genotype, with 56 isolates (87.5%), followed by L6 with seven isolates (10.9%) and L2 with one isolate (1.6%). L1, L4, L5, L7, and L8 were not detected. Among the 61 capsular type A isolates, 54 (88.5%) were L3 and seven (11.5%) were L6. Taken together, capsular and LPS typing results showed that A:L3 was the predominant circulating type among bovine *P. multocida* isolates in Hebei Province, accounting for 54 isolates (84.4%) of all isolates.

**Figure 2:**
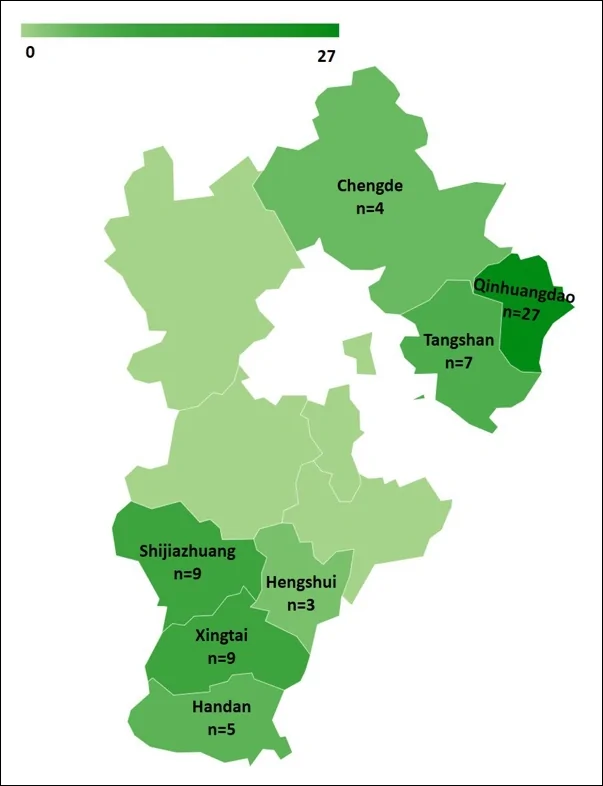
Geographic distribution of *Pasteurella multocida* isolates [Source: This map was created using Datawrapper and is available at https://app.datawrapper.de/fcxcbtmhll].

**Table 5:** Capsular and lipopolysaccharide typing profiles of Pasteurella multocida isolates (n = 64).

**Capsular type**	**L1**	**L2**	**L3**	**L4**	**L5**	**L6**	**L7**	**L8**	**Total (No.)**	**Percentage (%)**
A			54			7			61	95.3
B		1							1	1.6
D			1						1	1.6
E									0	0.0
F			1						1	1.6
**Total**		1	56			7			**64**	**100.0**

The percentage was calculated as (row or column total / 64) × 100% and rounded to one decimal place.

**Figure 3:**
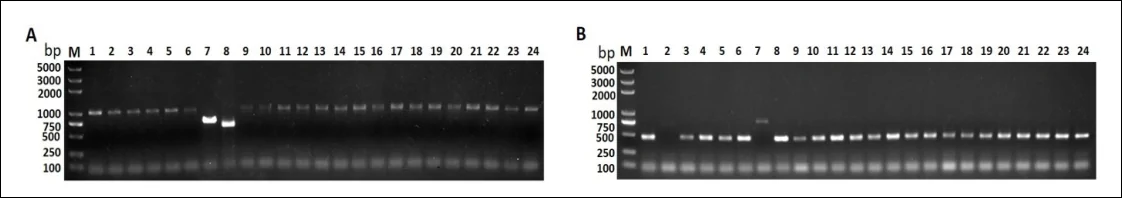
Partial identification results of capsular and LPS typing of *P. multocida* isolates. (A) Partial identification results of capsular typing of the isolates; (B) Partial identification results of LPS typing of the isolates.

### RIRDC MLST typing

The 64 *P. multocida* isolates were classified into eight sequence types (STs) based on amplification and sequencing of seven housekeeping genes and MLST analysis (Table 6). ST79 was the predominant type, comprising 51 isolates (79.7%). ST552 accounted for seven isolates (10.9%), whereas ST5, ST394, ST123, ST122, ST125, and ST9 were each represented by one isolate (1.6% each). Among capsular type A isolates, ST79 accounted for 83.6%. Among the predominant A:L3 isolates, ST79 accounted for 94.4% (51/54). Fisher's exact test indicated a strong association between ST79 and A:L3 (*p* < 0.0001, Φ = 0.8523), suggesting that this lineage represents the dominant clonal lineage of bovine *P. multocida* in Hebei Province.

**Table 6:** Distribution of RIRDC MLST sequence types among Pasteurella multocida isolates (n=64).

**Strain type**	**ST5**	**ST79**	**ST394**	**ST123**	**ST552**	**ST122**	**ST125**	**ST9**	**Total (No.)**	**Percentage**
A:L3	1	51	1	1					54	84.4
A:L6					7				7	10.9
B:L2						1			1	1.6
D:L3							1		1	1.6
F:L3								1	1	1.6
Total	1	51	1	1	7	1	1	1	64	100.0

The percentage was calculated as (row or column total / 64) × 100% and rounded to one decimal place. Fisher's exact test revealed a strong association between A:L3 and ST79 (p < 0.0001, Φ = 0.8523).

### Detection of virulence genes

Fifteen virulence genes from nine categories were detected in this study. The results showed that eight genes, *ompA*, *oma87*, *fimA*, *nanH*, *exbB*, *exbD*, *tonB*, and *hgbA*, had detection rates of 100% and were stably carried by all isolates (Figure 4). The *toxA* gene was not detected in any of the 64 isolates. The detection rates of *pfhA*, *tbpA*, *PmHAS*, and *tadD* were all 96.9%. The overall detection rates of *nanB* and *hgbB* were 23.4% and 17.2%, respectively. Notably, the detection rates of *nanB* and *hgbB* were only 13.7% and 7.8% in A:L3 isolates, respectively, whereas both genes were detected in 100% of A:L6 isolates. Fisher's exact test revealed significant differences in the carriage of both genes between A:L3 and A:L6 isolates (*p* < 0.0001), with *nanB* and *hgbB* being more prevalent in A:L6 (|Φ| = 0.6597 and |Φ| = 0.7676, respectively). This LPS genotype-associated difference suggests genetic differentiation among LPS types in adhesion, colonization, and iron-utilization capacity.

**Figure 4:**
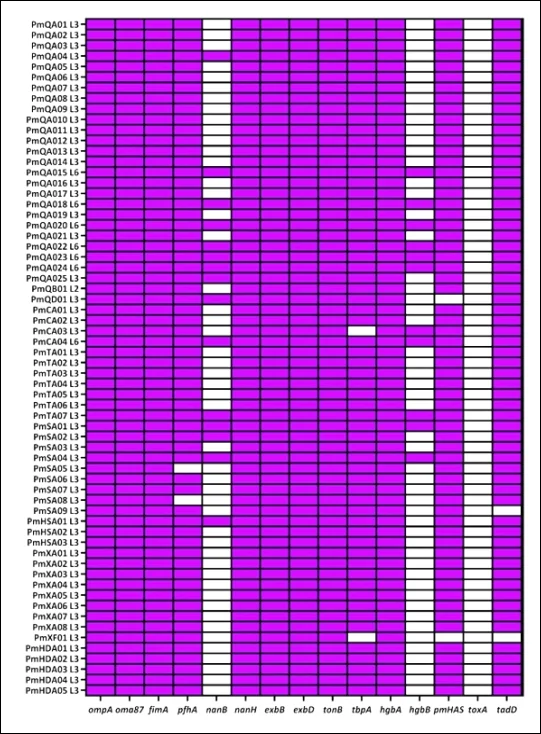
Virulence gene profiles of the isolates. Isolates are grouped by geographic origin; the letters "Q, C, T, S, HS, X, and HD" in strain names indicate the sample origins: Qinhuangdao, Chengde, Tangshan, Shijiazhuang, Hengshui, Xingtai, and Handan, respectively. Fisher's exact test revealed significant differences in the carriage of both *nanB* and *hgbB* between A:L3 and A:L6 isolates (*p* < 0.0001).

### Detection of antimicrobial resistance genes

PCR detection of 12 antimicrobial resistance genes from eight antimicrobial classes identified six resistance genes, whereas the remaining six genes were not detected. The macrolide resistance gene *mphE* had the highest detection rate, 92.2% (59/64), followed by the sulfonamide resistance gene *sul2*, 87.5% (56/64), making these the main resistance genes carried by the isolates (Table 7). The beta-lactam resistance gene *blaTEM* was detected in 35.9% (23/64) of isolates. The aminoglycoside resistance genes *strA* and *strB* were detected in 12.5% (8/64) and 9.4% (6/64) of isolates, respectively. The phenicol resistance gene *floR* was positive in only one isolate, with a detection rate of 1.6%. *ermA*, *tetA*, *qnrA*, *qnrB*, *blaOXA*, and *lnuA* were not detected.

**Table 7:** Distribution of antimicrobial resistance genes among the isolates (n = 64).

**Resistance gene class**	**Gene name**	**No. of positive isolates**	**Detection rate (%)**
Aminoglycosides	*strA*	8	12.5
Aminoglycosides	*strB*	6	9.4
Macrolides	*ermA*	0	0.0
Macrolides	*mphE*	59	92.2
Tetracyclines	*tetA*	0	0.0
Fluoroquinolones	*qnrA*	0	0.0
Fluoroquinolones	*qnrB*	0	0.0
Beta-lactams	*blaTEM*	23	35.9
Beta-lactams	*blaOXA*	0	0.0
Lincosamides	*lnuA*	0	0.0
Phenicols	*floR*	1	1.6
Sulfonamides	*sul2*	56	87.5

The detection rate (%) was calculated as (number of positive isolates / 64) × 100% and rounded to one decimal place.

### Antimicrobial susceptibility testing

The susceptibility of the isolates to 14 antimicrobial agents from eight classes was determined by the Kirby–Bauer method. The results (Figure 5) showed that the isolates were highly susceptible to doxycycline (DOX, 30 μg), enrofloxacin (ENR, 10 μg), ciprofloxacin (CIP, 5 μg), ceftiofur (CFT, 30 μg), and florfenicol (FFC, 30 μg), with susceptibility rates of 90.6-100%, and no or very few resistant isolates were detected for these agents. In contrast, the isolates showed generally high-level resistance to lincomycin (LIN, 2 μg), erythromycin (E, 15 μg), tylosin (TYL, 30 μg), and sulfadiazine (SUD, 300 μg), with resistance rates ranging from 93.8% to 100%. The overall susceptibility rates to tetracycline (TE, 30 μg), kanamycin (K, 30 μg), and gentamicin (GEN, 10 μg) were relatively high (>70%); however, isolates from Xingtai showed resistance rates exceeding 65% to all three agents, and Fisher's exact test revealed significant differences in resistance to these three antibiotics between Xingtai and other regions (*p* < 0.0001). Ceftriaxone (CRO, 30 μg) and ampicillin (AMP, 10 μg) showed relatively high intermediate rates (29.7% and 40.6%, respectively), but their resistance rates were low (<25%). Multidrug resistance analysis showed that 61 isolates were multidrug-resistant, corresponding to a multidrug resistance rate of 95.3% (Table 8). Thirty-five isolates (54.7%) were resistant to three antimicrobial classes, 14 (21.9%) to four classes, seven (10.9%) to five classes, and four (7.8%) to six classes. Resistance to three antimicrobial classes was the predominant multidrug resistance pattern.

### Pathogenicity assay

Three representative A:L3-ST79 isolates, QA-1, TA-1, and SA-1, were selected to determine *LD*₅₀ values in Kunming mice. All three isolates showed strong pathogenicity. Mice in the control group exhibited normal mental status, smooth and glossy fur, and normal body posture. Mice in the medium- and high-dose groups all developed obvious pathological symptoms, including rough and dull fur, weight loss, reduced activity, dyspnea, and lethargy. The *LD*₅₀ of QA-1 was 8.06 × 10¹ CFU, indicating extremely high virulence; the *LD*₅₀ values of TA-1 and SA-1 were 6.44 × 10³ CFU and 2.76 × 10³ CFU, respectively (Table 9). The clear differences in *LD*₅₀ among different isolates within the same clonal lineage suggest that virulence phenotypes may remain heterogeneous even when genotypes are consistent.

## DISCUSSION

In recent years, with the continued intensification and scaling-up of beef and dairy cattle production in China, stocking density has increased substantially, and the risk of respiratory disease and septicemia has also increased [27, 28]. As one of the important bacterial pathogens associated with bovine respiratory disease complex, *P. multocida* has become a key factor affecting herd health and the economic efficiency of cattle production [29]. In this study, the epidemiological characteristics, molecular types, virulence gene distribution, antimicrobial resistance profiles, and pathogenicity of bovine *P. multocida* isolates from Hebei Province were systematically analyzed. The results showed that capsular type A/LPS genotype 3 (A:L3) was the predominant circulating type in this region, and ST79 was the major sequence type, with a highly concentrated distribution among the predominant isolates. These findings are consistent with previous studies, in which capsular type A or LPS genotype L3 is commonly detected among *P. multocida* isolates associated with bovine respiratory disease (BRD) [30–33]. Meanwhile, ST79 is also one of the most frequently identified sequence types among bovine *P. multocida* isolates worldwide [33–35]. The present study indicates that this clonal lineage exhibits a distinct pattern of clonal expansion within Hebei Province, which is consistent with the global epidemic pattern of bovine *P. multocida*. This dominant clone may not only possess strong transmission potential but may also have certain advantages in host adaptation [12].

**Figure 5:**
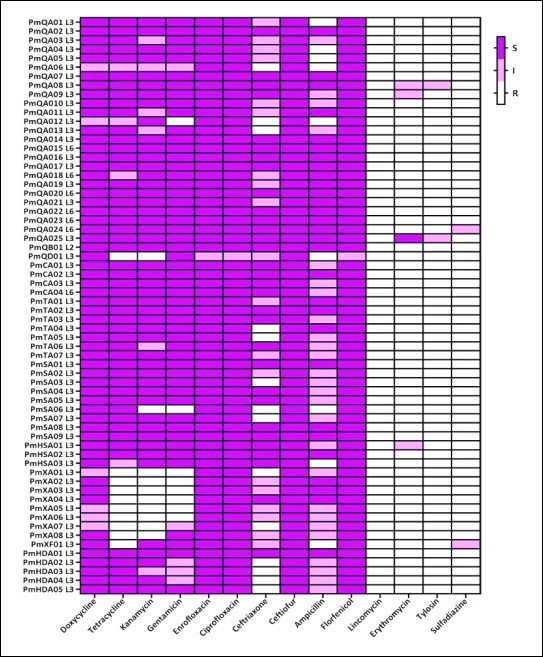
Antimicrobial resistance analysis of the isolates. Isolates are grouped by geographic origin; the letters "Q, C, T, S, HS, X, and HD" in strain names indicate the sample origins: Qinhuangdao, Chengde, Tangshan, Shijiazhuang, Hengshui, Xingtai, and Handan, respectively. Fisher's exact test revealed significant differences in the resistance rates to tetracycline, kanamycin, and gentamicin between isolates from Xingtai and those from other regions (p < 0.0001).

**Table 8:** Statistics of multidrug-resistant (MDR) isolates (n = 64).

**No. of resistance categories**	**No. of MDR isolates**	**Percentage**
3	35	54.7
4	14	21.9
5	7	10.9
6	4	7.8
Total	61	95.3

The percentage was calculated as (number of MDR isolates / 64) × 100% and rounded to one decimal place.

**Table 9:** LD₅₀ values of the isolates in Kunming mice.

**Strain**	**Challenge dose (CFU)**	**Deaths/total**	***LD*** **₅₀ (CFU)**
PmQA-1	6.3 × 10⁴	5/5	8.06 × 10¹
PmQA-1	6.3 × 10³	5/5	-
PmQA-1	6.3 × 10²	5/5	-
PmQA-1	6.3 × 10¹	2/5	-
PmQA-1	6.3 × 10⁰	0/5	-
PmTA-1	8.1 × 10⁵	5/5	6.44 × 10³
PmTA-1	8.1 × 10⁴	5/5	-
PmTA-1	8.1 × 10³	2/5	-
PmTA-1	8.1 × 10²	1/5	-
PmTA-1	8.1 × 10¹	0/5	-
PmSA-1	5.5 × 10⁵	5/5	2.76 × 10³
PmSA-1	5.5 × 10⁴	5/5	-
PmSA-1	5.5 × 10³	3/5	-
PmSA-1	5.5 × 10²	1/5	-
PmSA-1	5.5 × 10¹	0/5	-

The* LD*₅₀ was calculated using the Karber method according to the following formula: *LD*₅₀ = log⁻¹[Xm – i (∑p - 0.5)], where Xm is the logarithm of the highest dose, i is the interval between adjacent doses, and p is the mortality rate at each dose.

### Virulence gene profiles

Virulence gene detection showed that core virulence factors, including *ompA*, *oma87*, *fimA*, *nanH*, *exbB*, *exbD*, *tonB*, *hgbA*, *pfhA*, *tbpA*, *PmHAS*, and *tadD* were highly prevalent among the predominant isolates. These genes are primarily involved in key pathogenic processes, including bacterial adhesion, colonization, iron acquisition, and host invasion, indicating that circulating isolates in Hebei Province possess a relatively complete molecular basis for pathogenicity [3]. Among them, outer membrane proteins and fimbrial factors may facilitate early adhesion of the pathogen to the respiratory mucosa and persistent colonization, whereas iron acquisition-related genes may help the bacteria maintain growth and survival under iron-limited conditions in the host [1, 36]. Notably, the *toxA* gene was not detected in any of the isolates in this study. *toxA* encodes the *P. multocida* toxin (PMT), which is primarily associated with the development of progressive atrophic rhinitis (PAR) in pigs. This gene is sparsely distributed among *P. multocida* strains and is mainly found in type D and some type A isolates [17]. Bovine *P. multocida* primarily causes pneumonia and septicemia, with type A being the predominant serotype [37]; therefore, the absence of *toxA* in this study is consistent with this host–serotype association and aligns with previous reports on bovine isolates. In addition, although the overall carriage rates of *nanB* and *hgbB* in the isolates of this study differed significantly from the reports by Katsuda *et al.* and Ewers *et al.* [11, 17], they were respectively consistent with the findings of Verma *et al. *[12], Ujvári *et al. *[37], and Calderón *et al.* [38]. Both *nanB* and *nanH* genes encode sialidases; specifically, the NanH sialidase primarily hydrolyzes Neu5Acα (2→3) Gal, whereas NanB exhibits broad-spectrum sialidase activity capable of hydrolyzing both Neu5Acα (2→3) Gal and Neu5Acα (2→6) Gal [39]. The bovine respiratory tract, from the trachea to the alveoli, is predominantly enriched with Neu5Acα (2→3) Gal [40], and the NanH sialidase alone is sufficient to meet the nutritional requirements. Under long-term environmental selective pressure, the *nanB* gene may be lost, and the differential carriage of *nanB* and *nanH* observed in this study reflects the host-adaptive evolution of *P. multocida*. Both *hgbA* and *hgbB* genes encode hemoglobin-binding proteins; previous studies have demonstrated that the *hgbA* gene is widely distributed among *P. multocida* strains regardless of capsular type or host origin, whereas the prevalence of the *hgbB* gene varies with host origin and disease status [41, 42]. Furthermore, the significant differences in the carriage rates of *nanB* and *hgbB* among isolates with different LPS genotypes indicate that even within capsular type A isolates, substantial heterogeneity may exist in host preference, adhesion strategies, iron-utilization capacity, and virulence expression patterns [7].

### Antimicrobial resistance patterns

Antimicrobial resistance analysis further indicated that resistance among bovine *P. multocida* isolates from Hebei Province warrants close attention. In this study, the multidrug resistance rate reached 95.3%. *mphE* and *sul2* were the main resistance genes detected, generally consistent with the high resistance phenotypes observed to macrolides and sulfonamides. This result aligns with global resistance trends; moreover, additional resistance mechanisms to lincosamides beyond those identified in this study may exist in *P. multocida* [18, 43]. The high resistance rates observed for lincomycin, sulfadiazine, and related agents in this study are consistent with historical and ongoing patterns of lax regulatory oversight and easy access to these agents in veterinary practice. In China, these drugs were historically used as feed additives without prescription requirements until the nationwide withdrawal of growth-promoting drug feed additives in 2020 [44]. Furthermore, local enforcement of veterinary drug regulations has been fragmented, with little monitoring of antibiotic use or resistance in animal populations [45]. Globally, these agents share similar regulatory trajectories [46, 47]. The generic availability and low cost of these older antimicrobial classes further facilitate their widespread and often inappropriate use in intensive farming systems [48]. These factors collectively contribute to the continuous selective pressure that enriches resistant strains and promotes the dissemination of resistance genes such as *mphE* and *sul2* within *P. multocida* populations.

On the other hand, the high susceptibility observed for doxycycline, enrofloxacin, ceftiofur, and florfenicol in this study may be attributed to the strict regulatory oversight and prescription requirements for these agents in veterinary practice. These drugs are classified as veterinary prescription-only medicines or critically important antimicrobials with restricted use [49, 50], indicating that they may still retain reference value for clinical treatment when used legally and appropriately. The limited use of these agents in cattle production reduces selective pressure, thereby preserving their efficacy against *P. multocida*. Notably, the high resistance rates of Xingtai isolates to tetracycline, kanamycin, and gentamicin may be attributed to local antimicrobial use practices. National surveillance data on antimicrobial use in Chinese animal husbandry from 2018 to 2020 showed that tetracyclines, despite a declining trend, remained the most frequently used antimicrobial class, while the use of aminoglycosides increased significantly [51]. This long-term and non-standardized use of antimicrobials has created substantial selective pressure, promoting the dissemination of resistance genes among *P. multocida* populations. Furthermore, although regulatory oversight has been strengthened at the provincial level, challenges persist in grassroots enforcement, particularly in monitoring the off-label use of antimicrobial agents not approved for food-producing animals. To assess whether resistance profiles have expanded beyond approved veterinary drugs, ceftriaxone and ciprofloxacin were included in this study for *in vitro* resistance profiling and epidemiological comparison only. In addition, the genotypes and phenotypes of some antimicrobial agents were not fully consistent. For example, the detection rate of the β-lactam resistance gene *blaTEM* was higher than the ampicillin resistance rate, suggesting that resistance phenotypes may be influenced not only by the presence of resistance genes but also by factors such as gene expression levels and enzyme activity. Specifically, even when *blaTEM* is present, weak promoter activity leading to insufficient expression [52], or amino acid mutations in TEM-1 altering substrate specificity, may result in β-lactamase production or activity inadequate to hydrolyze clinically relevant concentrations of ampicillin [53]. In practice, antimicrobial treatment should be based on approved veterinary drugs, regional susceptibility monitoring, clinical indications, and withdrawal-period requirements, to achieve precise intervention while reducing the risk of further dissemination of resistance.

### Pathogenicity

Animal pathogenicity assays showed that the dominant A:L3-ST79 clone exhibited strong pathogenicity in the mouse model. In this study, the three representative isolates shared an identical core virulence gene profile; however, isolate SA-1 additionally carried the *nanB* and *hgbB* genes. Nevertheless, significant differences in *LD*₅₀ values were observed among the representative isolates, with QA-1 showing a lower *LD*₅₀ than both SA-1 and TA-1. This indicates that the additional carriage of *nanB* and *hgbB* did not confer enhanced virulence to SA-1, suggesting that virulence phenotypes are not simply determined by the number of virulence genes present but may be comprehensively regulated by factors such as gene expression levels, genomic background, and host response status [3, 5]. Therefore, carriage of virulence genes alone is insufficient to fully predict a strain’s actual pathogenicity, and comprehensive evaluation using animal models and phenotypic assays remains necessary.

### Public health and control implications

From a public health perspective, human infections with *P. multocida* are predominantly associated with bites or scratches from cats and dogs, and no confirmed cases of human infection with bovine *P. multocida* have been reported in the literature to date [54]. However, given the high multidrug resistance rate (95.3%) of the dominant A:L3-ST79 clone identified in this study and the potential risk of occupational exposure, continued surveillance of the molecular characteristics and antimicrobial resistance trends of bovine isolates remains important for One Health prevention and control strategies.

Based on these findings, prevention and control of bovine *P. multocida* in Hebei Province should focus on the predominant circulating clone. On the one hand, continuous surveillance of the A:L3-ST79 lineage should be strengthened to dynamically monitor its distribution, antimicrobial resistance changes, and virulence evolution. On the other hand, given the high prevalence, extensive multidrug resistance, and strong pathogenicity of this clone, vaccine development and immunoprophylaxis should be designed to target this major circulating type and its core virulence factor combinations, thereby improving the specificity and coverage of prevention and control strategies. Meanwhile, vaccination can significantly reduce antimicrobial use, alleviate selective pressure driving resistance from the source, decrease treatment costs and veterinary drug residue risks, and align with the One Health framework. In addition, antimicrobial use in clinical treatment should be further standardized, and regional, precision-based medication protocols should be formulated according to susceptibility testing results. These measures should be combined with improved feeding management, environmental control, and biosafety practices to minimize the disease risk associated with stress factors such as transportation, crowding, and mixed herding, thereby reducing the persistent transmission of the dominant clone in cattle populations [27, 29].

### Limitations and novelty

This study has several limitations. First, the samples were collected from only seven prefecture-level cities in Hebei Province and therefore may not fully represent the epidemiological situation across the entire province. Second, the study mainly relied on PCR detection of representative virulence and resistance genes and did not integrate whole-genome sequencing or transcriptomic data. Consequently, the evolutionary relationships, virulence regulatory networks, and resistance mechanisms of the predominant clone remain incompletely resolved. Future studies should combine whole-genome sequencing and RNA-seq to systematically elucidate the pathogenic mechanisms, resistance mechanisms, and adaptive evolution of the predominant clone at the genomic and transcriptional regulatory levels, thereby providing a stronger theoretical basis for precise surveillance, targeted prevention and control, and rational clinical medication for bovine respiratory disease.

The novelty of this study lies in its comprehensive, integrated characterization of bovine *P. multocida* in Hebei Province, the first of its kind in this key cattle-producing region. Unlike previous studies that typically focused on single aspects (e.g., serotyping or resistance alone), we combined molecular epidemiology (capsular/LPS + RIRDC MLST), virulence gene profiling, phenotypic/genotypic antimicrobial resistance analysis, and *in vivo* pathogenicity testing. This revealed the dominance of the A:L3-ST79 clonal lineage, high MDR prevalence, and strong pathogenicity, findings that significantly advance understanding of regional epidemiology and provide a foundation for targeted interventions. This work fills a critical knowledge gap in northern China and offers original insights into LPS genotype-associated virulence gene variation and the persistence of specific resistance genes under local antimicrobial use patterns.

## CONCLUSION

This study provides the first comprehensive characterization of bovine *P. multocida* isolates circulating in Hebei Province by integrating capsular and LPS typing, RIRDC MLST, virulence gene profiling, phenotypic and genotypic AMR analysis, and in vivo pathogenicity assessment. The findings demonstrated that the A:L3-ST79 clonal lineage predominated among the isolates and was characterized by high virulence and a high prevalence of MDR. Core virulence genes were highly conserved, whereas *toxA* was absent from all isolates. Although *mphE* and *sul2* were the predominant resistance genes and MDR was widespread, the isolates remained highly susceptible to doxycycline, enrofloxacin, ciprofloxacin, ceftiofur, and florfenicol, indicating that these agents may remain effective treatment options when supported by antimicrobial susceptibility testing.

From a practical perspective, identifying a dominant A:L3-ST79 lineage provides valuable epidemiological evidence to strengthen regional surveillance, improve outbreak tracing, support targeted vaccine development, and optimize antimicrobial stewardship in cattle production. The integrated characterization of molecular epidemiology, virulence, AMR, and pathogenicity offers a robust scientific basis for risk assessment and evidence-based control strategies against bovine pasteurellosis.

A major strength of this study is its comprehensive analytical approach, which combined multiple complementary molecular, microbiological, and pathogenicity assessments to provide a detailed understanding of the circulating *P. multocida* population in an important cattle-producing region of China. However, the study was limited to isolates collected from a single province and did not include whole-genome sequencing or transcriptomic analyses to further elucidate the genetic basis of virulence, host adaptation, and AMR. In addition, pathogenicity was evaluated only in a mouse model rather than in cattle, which may not fully reflect disease progression under natural host conditions.

Future studies should expand surveillance to other cattle-producing regions, incorporate WGS-based phylogenomic analyses, investigate the mobile genetic elements responsible for AMR dissemination, and evaluate the immunogenicity and protective efficacy of vaccines targeting the predominant A:L3-ST79 lineage. Longitudinal monitoring of circulating clones and resistance trends will also be essential to detect emerging variants and guide timely interventions.

Overall, these findings establish an important epidemiological baseline for bovine *P. multocida* in Hebei Province and provide valuable evidence to support precision surveillance, rational antimicrobial use, vaccine development, and sustainable prevention and control programs for bovine pasteurellosis.

## DATA AVAILABILITY

The supplementary data can be obtained from the corresponding author upon reasonable request.

## AUTHORS’ CONTRIBUTIONS

YTL, JJH, JLP, and XQZ: Collected the samples, performed the laboratory investigations, curated the data, conducted the formal analysis, interpreted the data, validated the results, and drafted the manuscript. LC, ZQZ, YHL, XCP, and ZTL: Interpreted the data, validated the results, and critically reviewed the manuscript. TLW and QMS: Conceived and designed the study, supervised the research, interpreted the data, critically revised the manuscript, and acquired funding. All authors have read and approved the final manuscript.
